# An Overview of the Role of Lipofuscin in Age-Related Neurodegeneration

**DOI:** 10.3389/fnins.2018.00464

**Published:** 2018-07-05

**Authors:** Alexandra Moreno-García, Alejandra Kun, Olga Calero, Miguel Medina, Miguel Calero

**Affiliations:** ^1^Chronic Disease Programme-CROSADIS, Instituto de Salud Carlos III, Madrid, Spain; ^2^Biochemistry Section, Science School, Universidad de la República, Montevideo, Uruguay; ^3^Protein and Nucleic Acids Department, Instituto de Investigaciones Biológicas Clemente Estable, Montevideo, Uruguay; ^4^Centro de Investigación Biomédica en Red sobre Enfermedades Neurodegenerativas, Madrid, Spain; ^5^Alzheimer Disease Research Unit, CIEN Foundation, Queen Sofia Foundation Alzheimer Center, Madrid, Spain

**Keywords:** aging, amyloid, autofluorescence, lipofuscin, neurodegeneration, oxidative stress, protein deposits

## Abstract

Despite aging being by far the greatest risk factor for highly prevalent neurodegenerative disorders, the molecular underpinnings of age-related brain changes are still not well understood, particularly the transition from normal healthy brain aging to neuropathological aging. Aging is an extremely complex, multifactorial process involving the simultaneous interplay of several processes operating at many levels of the functional organization. The buildup of potentially toxic protein aggregates and their spreading through various brain regions has been identified as a major contributor to these pathologies. One of the most striking morphologic changes in neurons during normal aging is the accumulation of lipofuscin (LF) aggregates, as well as, neuromelanin pigments. LF is an autofluorescent lipopigment formed by lipids, metals and misfolded proteins, which is especially abundant in nerve cells, cardiac muscle cells and skin. Within the Central Nervous System (CNS), LF accumulates as aggregates, delineating a specific senescence pattern in both physiological and pathological states, altering neuronal cytoskeleton and cellular trafficking and metabolism, and being associated with neuronal loss, and glial proliferation and activation. Traditionally, the accumulation of LF in the CNS has been considered a secondary consequence of the aging process, being a mere bystander of the pathological buildup associated with different neurodegenerative disorders. Here, we discuss recent evidence suggesting the possibility that LF aggregates may have an active role in neurodegeneration. We argue that LF is a relevant effector of aging that represents a risk factor or driver for neurodegenerative disorders.

## Introduction

Despite aging being by far the greatest risk factor for highly prevalent neurodegenerative disorders, the molecular underpinning of age-related brain changes is still not well understood (Kukull et al., 2002). Physiological changes associated with aging are inherent to all animals, representing an extremely complex and multifactorial process involving the simultaneous interplay of several processes operating at many levels of the functional organization.

Recently, it has been proposed a classification of the main cellular and molecular events affected by aging, and particularly by brain aging (López-Otín et al., 2013). For any factor to be considered a hallmark of aging, it should meet the following criteria: (I) it should be present during normal aging; (II) its exacerbation should trigger an accelerated aging; and (III) its amelioration should prevent the normal aging course, even extending lifespan. Accordingly, one of the most relevant features of aging is related to the increasingly dysfunctional mechanisms of renewal of cellular constituents that precludes the clearance of damaged biomolecules and organelles and its replacement by new functional structures. This sustained inefficient recycling mechanism leads to the accumulation of unfit molecules that further interfere with cellular functions, preferentially within long-lived post-mitotic cells such as neurons (Hung et al., 2010; López-Otín et al., 2013). Among the main components of this biological “garbage,” we could find indigestible protein aggregates, defective mitochondria and lipofuscin (LF) (Terman, 2001).

LF is a post-mitotic pigment traditionally associated with aging (“age pigment”) (Terman and Brunk, 1998). In this review, we discuss recent evidence that suggests the possibility that rather than being a mere bystander of the aging process, LF may have an active role in neurodegenerative disorders such as Alzheimer's disease (AD), Parkinson's disease (PD), age-related macular degeneration (AMD).

## Lipofuscin composition and distribution

LF is a fluorescent complex mixture composed of highly oxidized cross-linked macromolecules (proteins, lipids, and sugars) with multiple metabolic origins (Höhn et al., 2010; König et al., 2017; Rodolfo et al., 2018). The nature and structure of LF complexes seem to vary among tissues and show temporal heterogeneity in composition of oxidized proteins (30–70%), lipids (20–50%), metals cations (Fe^3+^, Fe^2+^, Cu^2+^, Zn^2+^, Al^3+^, Mn^2+^, Ca^2+^) (2%), and sugar residues (Benavides et al., 2002; Double et al., 2008).

Because of its polymeric and highly cross-linked nature, LF cannot be degraded, nor cleared by exocytosis, thus being accumulated within the lysosomes and cell cytoplasm of long-lived post-mitotic and senescent animal cells. Opposite, proliferative cells efficiently dilute LF aggregates during cell division, showing low or no accumulation of the pigment (Brunk and Terman, 2002; Porta, 2002; Terman and Brunk, 2005; Rodgers et al., 2009; Firlag et al., 2013). For this reason, LF deposits are especially abundant in nerve cells, cardiac muscle cells, and skin.

LF fluorescence shows a large heterogeneity in the emission spectra, which reveals differences in its chemical composition, as a result of its ripening in specific metabolic pathways (Schwartsburd, 1995). In general, the LF fluorescence emission presents a very wide spectra ranging from 400 to 700 nm, with a maximum around of 578 nm for excitation at 364 nm (Warburton et al., 2007). Due to its elevated levels within brain tissue, LF fluorescence interferes with different analytical techniques such as immunoconfocal microscopy. Thus, different experimental protocols have been used to block LF autofluorescence in tissue samples, such as Sudan Black, copper sulfate or picric acid treatments. However, these methods do not allow the study of LF concurrently with neurodegeneration markers, thus impeding the analysis of LF contribution to pathology.

**Figure 1:**
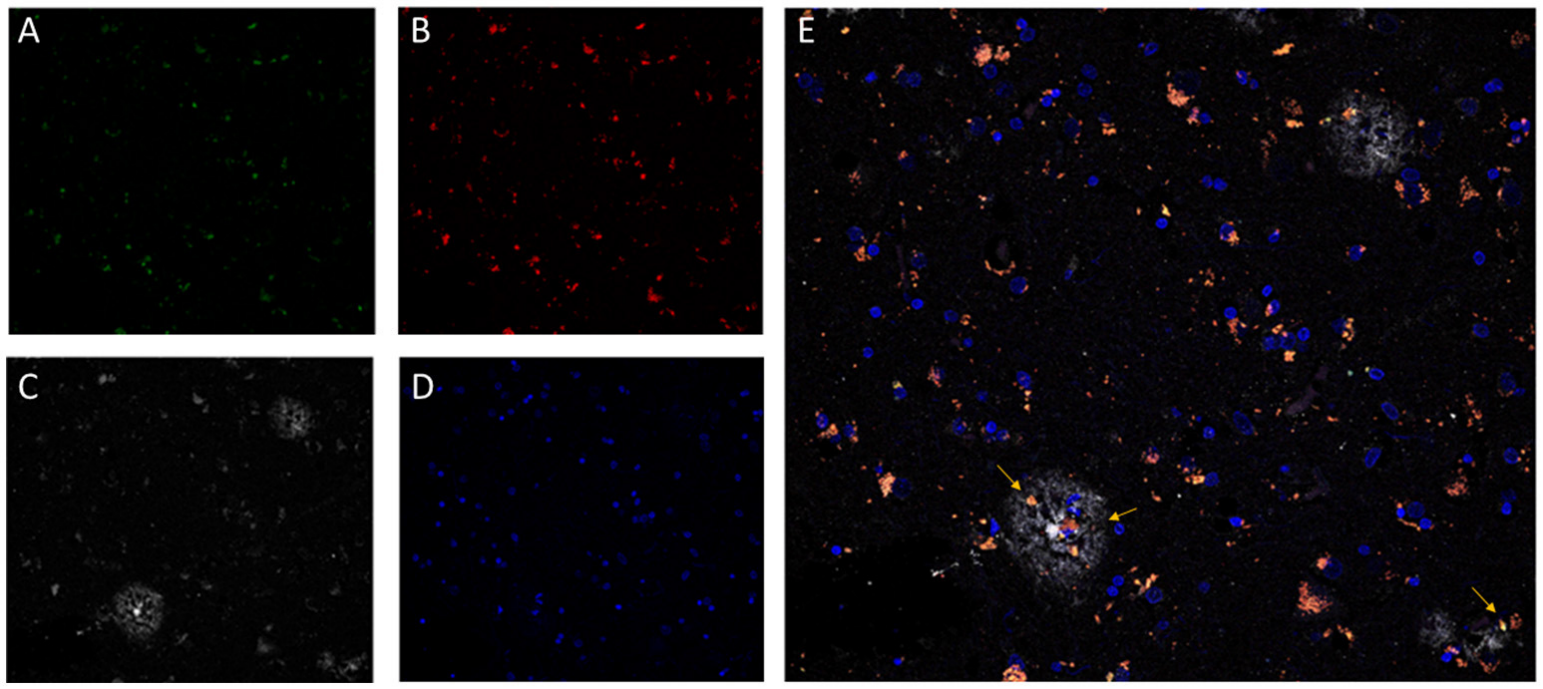
Confocal fluorescence microscopy analysis of AD brain tissue. The characteristic perinuclear lipofuscin deposits can be clearly identified in brain tissue by autofluorescent emission at 510–530 nm (**A**, green) and at 570–600 nm (**B**, red), with excitation at 488 nm and 561 nm, respectively. Additionally, amyloid beta plaques (**C**, white) were immunostained by the specific monoclonal antibody 4G8 (Covance) followed by an anti-antibody conjugated to a fluorophore excitable to 633 nm and emitting light from 670 to 700 nm (Invitrogen GAM-A-21052). DNA in nuclear domains was identified by DAPI probe (**D**, blue). In the merged image **(E)**, lipofuscin aggregates (pink-orange) appears widely distributed throughout the tissue with incidental colocalization within the amyloid beta plaques. Images represent a single confocal plane of cryosections of prefrontal cortex from an AD patient treated with 70% formic acid for 10 s. The yellow arrows indicate LF aggregates that are located within senile plaques. Adapted with permission from chapter 31 “Characterization of Amyloid-β Plaques and Autofluorescent Lipofuscin Aggregates in Alzheimer's Disease Brain: A Confocal Microscopy Approach” in Amyloid Proteins. Methods and Protocols, Volume 1179 in Methods Molecular Biology Series (ISBN: 978-1-4939-7815-1); Series Ed.: Walker, John M. Humana Press-Springer.

Additionally, as LF plays a clear role in cellular senescence, and increasing interest is focused on the study of its potential pathophysiological role, several methods have been developed to quantify LF in brain tissue. Based on its high lipid content, classical methods of isolation and quantitation of LF employed organic solvent extraction or density gradient ultracentrifugation protocols (Siakotos, 1974; Taubold et al., 1975; Ottis et al., 2012). However, due to its fluorescent properties, most recent methods for LF detection and quantification are based on the use of fluorescence microscopy (Moore et al., 1995; Jung et al., 2010; Zheng et al., 2010; Jensen et al., 2016). Provided that LF presents a very broad fluorescence spectrum, fluorescence images of tissue preparations can be acquired over a broad range of wavelengths. Depending on the need to colocalized LF autofluorescence with other cellular structures, non-overlapping anti-antibodies or probes should be selected (Jung et al., 2010). For example, DNA probes emitting in the far-red range can be combined with the detection of LF in both green and red channels (Zheng et al., 2010). Recently, we have developed a method based on channel filtering of confocal microscopy to identify and discriminate LF autofluorescence signals from the specific ones, such as amyloid plaques in the AD brain (see Figure 1) (Kun et al., 2018).

## Mechanisms of lipofuscin accumulation

### Altered cellular proteostasis

Three main mechanisms actively maintain cellular proteostasis: (i) degradation by the ubiquitin-proteasome system (UPS); (ii) re-folding and suppression of aggregates by heat shock proteins; and (iii) clearing of abnormal cell contents by lysosome-mediated autophagy (macroautophagy) (Mizushima et al., 2004; Mizushima, 2007). Both under normal and pathological conditions, the combination of these pathways are also possible (Fortun et al., 2003, 2005; Hara et al., 2006), including chaperone-mediated autophagy (Kaushik and Cuervo, 2012) and the ubiquitin-mediated autophagy required for the UPS system (Opalach et al., 2010).

With aging, cellular proteostasis becomes less effective, hampering degradation of misfolded proteins, which in turn expose their hydrophobic domains and tend to form high-order complexes with other perinuclear/centrosomal-proximal proteins that are prone to aggregate into aggresomes (Kettern et al., 2011; Richter-Landsberg and Leyk, 2013; Seiberlich et al., 2013; Popovic et al., 2014; An and Statsyuk, 2015; Rodolfo et al., 2018). Then, upon lysosomal uptake of these aggregates via macroautophagy, highly cross-linked materials such as LF accumulate within the lysosomes (Höhn and Grune, 2013). Thus, although most of intracellular LF is located in the lysosomes, after inhibition of the macroautophagy pathway, LF can accumulate also in the cytosol (Höhn and Grune, 2013).

These authors propose a mechanism by which the presence of high amounts of LF inhibit the proteasome, preventing the degradation of oxidized proteins and increasing the formation of free radicals, leading to cytotoxicity (Sitte et al., 2000; Ryhänen et al., 2009; Höhn et al., 2011). Then, oxidized proteins further accumulate and tend to aggregate (Höhn and Grune, 2013; Reeg and Grune, 2015). Hence, the 20S proteasome binds to the surface of LF aggregates at patches rich in unfolded oxidized proteins, but it remains attached, unable to degrade the LF due to its cross-linked nature (Reeg and Grune, 2015). In turn, lysosomal LF appears to impair autophagy and lysosomal degradation, resulting in increased reactive oxygen species (ROS) generation, protein oxidation, further aggregation, and further LF formation (Terman and Brunk, 2004). Moreover, cytosolic LF deposits produce ROS, decreasing cellular viability by defective cytosolic protein degradation (Grune et al., 2001; Höhn and Grune, 2013; Lei et al., 2017).

In healthy conditions, the functional cost of proteostasis is high, but essential (Rangaraju et al., 2009; König et al., 2017). A consequence of the inhibition of the proteasome system is the decreased degradation of pro-apoptotic proteins (including c-jun, Bax, and p27), triggering the initiation of the apoptotic cascade (Powell et al., 2005), as well as the blockage of NFkB nuclear translocation and signal transduction (Powell et al., 2005; Reeg and Grune, 2015). Thus, cells overloaded with cytosolic and lysosomal LF may become dysfunctional and undergo apoptotic proteostasis-mediated cell death (Powell et al., 2005; Höhn et al., 2011).

Additionally, LF ability to incorporate different metals contributes to reactive oxygen species (ROS) generation through the Fenton reaction and further protein oxidation and LF formation (Jolly et al., 1995; Tokutake et al., 1995; Reeg and Grune, 2015). For example, in Mn^2+^-exposed wild-type mice, it was observed an increased number of LF granules associated with neurodegenerating spiral ganglion neurons (Ohgami et al., 2016). Similarly, in ferritin light chain gene transgenic mice, it was shown the accumulation of LF granules within iron deposits, particularly at degenerating regions of the cerebellum and striatum (Maccarinelli et al., 2015).

### Mitochondrial involvement in lipofuscinogenesis

The oxidative stress and the accumulated mitochondrial DNA mutations during lifetime lead to impairment metabolism of mitochondria, which in turn yields further oxidative stress by ROS production through oxidative phosphorylation. Cellular quality control mechanisms activate mitophagy to eliminate non-functional mitochondria by the lysosomal system (Atkins et al., 2016; Rodolfo et al., 2018), and then, the lysosomes accumulate non-degraded molecules, such as the mitochondrial small hydrophobic ATP-synthase subunit-c, which appears to be the main component of LF in neuronal ceroid lipofuscinosis diseases (Ezaki et al., 1995; Vidal-Donet et al., 2013). During aging, mitochondrial repair systems are further compromised by a general reduction of macroautophagy and downregulation of specific mitochondrial proteases, which are responsible for the degradation of oxidatively modified proteins (Ngo and Davies, 2007; Davies, 2014; König et al., 2017).

These findings underscore an important crosstalk between mitochondria and lysosomes during aging that directly involves LF and other macromolecular aggregates (Terman et al., 2010). Accumulation of LF within the lysosomes leads to reduced autophagy and lower turnover of effective mitochondria. In turn, functionally deficient mitochondria generate increased ROS levels that further intensifies lipofuscinogenesis in a feedback loop of dysfunctional mitochondria and lysosomes, resulting in enhanced oxidative stress, lower energy production and dysfunction of the catabolic pathways (Brunk and Terman, 2002; Terman et al., 2010).

### Lipid metabolism

As lipids are one of the main components of LF, a dysfunction of lipid metabolism has been suggested for the synthesis and accumulation of LF. Thus, Hebbar and collaborators have proposed that the buildup of LF deposits is a direct consequence of an imbalance in brain ceramides and sphingosines at early stages of neurodegeneration (Hebbar et al., 2017). Zhao and collaborators also demonstrated that defective ceramide biosynthesis causes neurodegeneration, suggesting a novel mechanism for LF formation (Zhao et al., 2011). Similarly, Pan and collaborators demonstrated in an *in vivo* knock-out mouse model that neuraminidases 3 and 4 play a key role in the central nervous system (CNS) function through the catabolism of gangliosides, and the prevention of their conversion into LF aggregates (Pan et al., 2017). Additionally, Horie and collaborators demonstrated that LF is also constituted by glycation products which interact through Schiff base reactions with protein-lipid complexes (Horie et al., 1997).

The various mechanisms of production and accumulation of LF discussed within this section depict a complex panorama in which the lysosomes play a central role in lipofuscinogenesis. Thus, the increasing amount of LF deposits during aging in certain post-mitotic tissues, and the massive buildup of LF in disorders associated with lysosomal dysfunction, such as *Neuronal Ceroid Lipofuscinosis* (see next sections), are arguably some of the best established findings about the pathophysiological accumulation of LF. However, due to its diverse origin, amalgamated composition, cross-linked nature, autofluorescent properties, and its age-related ubiquitous distribution within the CNS, the role of LF in neurodegeneration is still yet to be elucidated. Moreover, the analysis of a potential pathophysiological role of LF has been hampered by the absence of adequate animal models and their corresponding controls; thus, underscoring the need for simpler system study models.

## *In vitro* lipofuscin synthesis for neurodegenerative studies

In order to explore the physicochemical properties, interactions, and functions of LF, it is essential to have a reliable system to produce it, either *in vitro* or in *in vivo* models. Numerous authors have described different approaches to obtain LF from diverse biological sources. For example, several methods have been established to produce N-retinylidene-N-retinylethanolamine (A2E), which is one of the principal fluorescent components of LF from retinal pigmented epithelial cells (Parish et al., 1998).

Other authors, considering that LF is the final product of a peroxidation reaction between lipids and proteinaceous components within the cell, have used the process of photo-oxidation of subcellular fractions to obtain high quantities of “synthetic” LF through UV irradiation (Nilsson and Yin, 1997; Höhn et al., 2010; Frolova et al., 2015). Interestingly, these studies demonstrate that mitochondria can produce LF granules without oxidative factors (oxygen saturation or pro-oxidants) and that the presence of lipids is not an absolute requirement for LF formation (Frolova et al., 2015). These methods allow the synthesis of LF similar to that found in post-mitotic cells with analogous composition and properties. However, for some experimental setups, naturally produced LF may be more suitable and relevant. Arguably, LF fractions purified from the retinal pigment epithelium (RPE) or derived from cell culture models through organic solvent extractions are the most widely used procedures (Folch et al., 1957; Lamb and Simon, 2004; Boulton, 2014; Feldman et al., 2015).

## Lipofuscin in neurodegeneration

As mentioned above, LF is considered a hallmark of cellular aging. In fact, the accumulation with time of LF pigments within post-mitotic cells is so constant that it is used to calculate the age of crustacean (Pearse, 1985; Maxwell et al., 2007). In normal aged mammal brains, LF distributes delineating a specific senescence pattern that correlates with altered neuronal cytoskeleton and cellular trafficking. Thus, as we age, the brain of the human adult becomes heavily laden with intraneuronal deposits of LF and neuromelanin pigment (Braak et al., 1999). However, in neurodegenerative disorders, LF aggregates appear to increase not only with age but also with pathological processes such as neuronal loss, proliferation, and activation of glial cells, and a repertoire of cellular alterations, including oxidative stress, proteasome, lysosomal, and mitochondrial dysfunction (González-Scarano and Baltuch, 1999; Grune et al., 2004; Keller et al., 2004; Riga et al., 2006a; Hebbar et al., 2017; Wellings et al., 2017).

### Neuronal ceroid lipofuscinosis

Arguably, the most relevant group of disorders clearly related with LF deposits are the neuronal ceroid lipofuscinoses (NCLs). NCLs are a group of rare neurodegenerative disorders characterized by intracellular (and at a minor extent extracellular) accumulation of a fluorescent lipopigment called “ceroid lipofuscin” (Kohan et al., 2011). Clinically, NCLs are associated with variable, progressive symptoms, including dementia, visual loss, seizures, and cerebral atrophy (Grubman et al., 2014). Genetically, NCLs are a homogeneous group associated with mutations in at least 14 affected genes termed as from *CLN1* to *CLN14* generally with autosomal recessive inheritance (for an updated list of mutation check http://www.ucl.ac.uk/ncl/mutation.shtml). Mutations in these genes are associated with several distinct NCL disorders, which share several common features, including inflammation, lysosomal LF deposits, and lipid abnormalities, but have different age at onset, clinical phenotypes, and progression rates (Grubman et al., 2014). In general, NCLs are considered lysosomal storage diseases; although, they also exhibit characteristics that set them apart from typical lysosomal storage diseases (Mink et al., 2013; Nita et al., 2016). One prototypical example of these disorders is NCL2. The NCL2 disease is one type of inherited infantile neuronal ceroid lipofuscinoses caused by the deficiency of the lysosomal enzyme tripeptidyl peptidase 1, which affects the brain and the retina and is characterized by the lysosomal accumulation of ceroid LF (Williams et al., 2017). Interestingly, related to the presence of “normal” LF aggregates in tissue, the diagnosis of adult forms of NCL is challenging due to the misinterpretation of age-related LF deposits as abnormal storage material, with more than one-third of cases misdiagnosed; therefore, stressing the need of analysis of known causative genes and the identification of newly associated genes (Berkovic et al., 2016).

Outside this group, several other disorders show resemblance with the NCLs as they progress with ceroid LF deposition. Among them, we highlight disorders that are associated with mutations in the *CLC-3* (a member of the CLC chloride channel family) gene (Yoshikawa et al., 2002), the *ClC-7* (chloride channel of late endosomes and lysosomes) gene (Kasper et al., 2005; Weinert et al., 2010), the *cathepsin D* (a major lysosomal protease) gene (Koike et al., 2000), as well as neuronopathic osteopetrosis associated to defects in the genes *TCIRG1* or *ClC-7* (Steward, 2003). In these disorders, far from being a consequence of the neurodegenerative process, LF appears to have an important role in the etiology of clinical symptoms.

### Age-related macular degeneration

Age-related macular degeneration (AMD) is a degenerative disorder of the RPE of the central retina, which represents the most important cause of vision loss in the elderly. It has a multifactorial etiopathology and is characterized by the presence of deposits of lysosomal origin, such as LF, melanolipofuscin, and melanosomes (Rodríguez-Muela et al., 2013; Pollreisz et al., 2018). It is generally accepted that LF can contribute to the pathogenesis of AMD. Due to its nature as a fluorochrome, exposure of the spherical microparticles of LF at the RPE to blue light produces a phototoxic and proinflammatory effect, mediated by reactive oxygen intermediates (ROI), which contributes to atrophy in AMD patients (Brunk and Terman, 2002; Pollreisz et al., 2018). Interestingly, A2E, a major LF fluorophore that accumulates during AMD progression, appears to be directly involved in the photo-induced oxidative stress, and the disruption of membrane integrity at the RPE (Lamb and Simon, 2004). Similarly, the levels of A2E in the retina of an AMD mouse model [Ccl2(-/-)/Cx3cr1(-/-) mice] were significantly higher when compared to wild-type retina (Tuo et al., 2007).

In addition, Stargardt disease, the most common form of inherited juvenile macular degeneration which is associated with progressive vision loss caused by the death of photoreceptor cells in the macula of the retina, presents significant LF accumulation in the lysosomal compartment of the retinal pigment epithelium of the eye (Adler et al., 2015).

Furthermore, LF particles have been recently detected in the aged human optic nerve from controls, and glaucoma or AMD donors (de Castro et al., 2013). Although similar amounts of LF were observed among the different groups, the authors found that optic nerves derived from donors with glaucoma contain larger LF particles compared to those observed in the age-matched control and AMD groups. Moreover, they observed that optic nerves from glaucoma donors display a smaller diameter and higher concentration of LF relative to age-matched controls, suggesting that the increased LF levels may have an active role in inducing pro-inflammatory phenotypes in microglia and astrocytes, thus exacerbating optic nerve damage (de Castro et al., 2013).

### Frontotemporal degeneration

Heterozygous loss-of-function mutations in the progranulin gene (*GRN*) are associated with frontotemporal degeneration with TDP-43-positive inclusions (FTD-TDP). FTD is highly heterogeneous in its clinical presentation, age at onset and duration of the disease even within the same families, suggesting the mediation of additional modifying genes. Strikingly, while the deletion c.813_816del (rs63749877) in the *GRN* gene in heterozygosis is also associated with FTD, the same mutation when presented in homozygosis causes adult-onset NCL, revealing a remarkable link between neurodegeneration and lipofuscinogenesis (Smith et al., 2012). More recently, Ward and collaborators have demonstrated that heterozygous *GRN* mutation carriers also present preclinical retinal lipofuscinosis, as well as increased lipofuscinosis and intracellular NCL-like deposits in the cerebral cortex (Ward et al., 2017).

In animal models, progranulin deficiency partially recapitulates FTD behavioral abnormalities and neuropathological changes, including altered dendritic morphology and synaptic deficits in the hippocampus (Petkau et al., 2016). Aged progranulin-deficiency mice also show increased lipofuscinosis, microgliosis, and astrogliosis, as well as mild regional specific cell loss. However, conditional loss of progranulin in neurons is not sufficient to cause neuronal ceroid lipofuscinosis-like neuropathology in mice, as progranulin expressed by the microglia appears to compensate the neuronal deficit of progranulin (Petkau et al., 2017). Interestingly, although neurodegeneration and LF accumulation clearly correlate in most animal models, in this progranulin deficiency mouse model, it has been shown that deletion of the *TMEM106B* (Transmembrane protein 106B) gene normalizes lysosomal activity and rescues FTD-related behavioral abnormalities and retinal degeneration, without showing a decrease in LF accumulation (Klein et al., 2017); therefore, suggesting that neurodegeneration and lipofuscinogenesis are independent processes. As recent evidence suggests that progranulin is a key player for the appropriate maintenance of lysosomal function during aging (Zhou et al., 2017), these data point out to a common role of endolysosomal dysfunction for FTD neurodegeneration and NCL pathology. Further studies should be aimed at elucidating the specific pathological role LF in FTD neurodegeneration.

### Parkinson's disease

In PD, LF granular aggregates and upregulation of α-synuclein (α-syn) appear to be directly involved in the selective degeneration of dopaminergic neurons in the SNC (Lv et al., 2011). Additionally, increased expression of divalent metal transporter 1 and decreased expression of ferroportin 1 seem to associate with elevated levels of iron at the SNC; therefore, suggesting that increased iron levels are involved in the selective degeneration of dopaminergic neurons (Lv et al., 2011). Interestingly, Braak and collaborators found that only those neuronal types containing LF or neuromelanin show an increased susceptibility to develop pathological changes including cytoskeletal changes and α-syn Lewy body deposits (Braak et al., 2001). Moreover, α-syn appears to be a constituent of the lipofuscin deposits present in the neurons of the *substantia nigra* of PD patients and in nigral neurons of MPTP-treated mice, suggesting that LF deposits may have a role in the selective nigral degeneration (Braak et al., 2001; Meredith et al., 2002).

Similarly, in Deiters' neurons, which express the cytostructural non-phosphorylated neurofilament protein (NPNFP), Wellings and collaborators found a strong inverse correlation between NPNFP intensity and LF autofluorescence, suggesting that these changes contribute to degeneration of postural reflexes observed in PD (Wellings et al., 2017). According to the authors, LF accumulation may lead to cytoskeletal abnormalities, including decreased NPNFP levels and disrupting Deiters' neurons functions, even in neuropathological normal controls (Wellings et al., 2017). Interestingly, non-dopaminergic areas as the medullary nuclei that are early affected during PD progression (Braak et al., 2001) present concomitant accumulation of Lewy bodies and LF granules (Wellings et al., 2017). Similarly, Braak and collaborators described that Lewy bodies are normally found within the LF deposits of medullary neurons (Braak et al., 2003). Opposite, Drach and collaborators found reduced intraneuronal LF content in patients with Lewy body dementia (LBD) compared to AD and controls, arguing that these findings are related to either a reduced metabolic rate in LBD or to an uneven use of neuroleptics among groups (Drach et al., 1998).

### Alzheimer's disease

In AD, at initial stages of neurodegeneration, affected pyramidal neurons in prefrontal cortex and hippocampus increasingly accumulate lysosomes at the basal pole and then extensively in the perikarya and proximal dendrites (Cataldo et al., 1994). Lysosomes become filled with LF aggregates as a result of initial autophagocytosis and lysosomal system activation. At more advanced stages of neurodegeneration, surviving neurons show a great cytoplasmic accumulation of large LF deposits. Interestingly, following neuronal death, LF aggregates are found in the extracellular space, usually associated with senile β-amyloid plaques (Cataldo et al., 1994). Tokutake and collaborators suggested that LF granules associated to the senile plaques are the responsible for binding aluminum, providing an argument to explain the divergent reports on the presence of this metal in AD plaques (Tokutake et al., 1995).

LF is formed when products of lipid peroxidation (reactive aldehydes and lipid radicals) attack lipids, proteins and other susceptible groups, either locally or far from their origin, because they are diffusible and can migrate and act at a distance. Consequently, lipid peroxidation end-products (also called LF-like pigments), are produced and their distribution could be either restricted to a specific tissue or tissue domains or be systemically distributed. For instance, in brains from AD patients, the lipid peroxidation product malondialdehyde, an important component of the phospholipid bilayer membranes of neurons, is significantly increased (Dalle-Donne et al., 2006); thus, promoting free radical damage, protein cross-linking and decreased membrane fluidity (Goldstein and McDonagh, 1976; Chmátalová et al., 2016). Thus, the reactive compounds from brain tissue can act like a messenger, diffusing through the bloodstream and attacking polyunsaturated fatty acids in membranes of erythrocytes or soluble plasma proteins leading to the formation of LF. In AD patients, it was found that erythrocyte-bound LF levels were significantly higher than those observed in controls (Goldstein and McDonagh, 1976; Skoumalová and Hort, 2012).

In the same line, in the cerebral cortex of aged control and AD brains, a subset of neurons covered by perineuronal nets (PNs) are less frequently affected by LF accumulation. The polyanionic character of PNs appears to contribute to reducing local oxidative potential in the neuronal microenvironment by scavenging and binding redox-active iron (Morawski et al., 2004). Braak and Braak previously observed a similar effect related to cortical myelination. They found a clear inverse correlation between the average myelin content and the density of intraneuronal LF deposits, as well as with the appearance of the neurofibrillary changes associated with neurodegeneration, suggesting that the LF-laden neurons with a long, thin, and sparsely myelinated axon are prone to develop AD-related changes (Braak and Braak, 1998). Interestingly, LF deposits are also found to colocalize with β-amyloid plaques (Riga et al., 2006a; Moreira et al., 2010; Firlag et al., 2013). Altogether, these facts have set the basis for the hypothesis that the release of LF into the extracellular space following the death of neurons may substantially contribute to the formation of AD senile plaques and neurodegeneration (Giaccone et al., 2011).

### Huntington disease

Huntington's disease (HD) is an inherited disorder caused by an autosomal dominant mutation in the Huntingtin gene (*htt*), related to the expansion of CAG triplet repeats, resulting in an extended polyglutamine stretch (polyQ) within the huntingtin protein, which becomes unstable, gradually damaging neuronal cells (Ross et al., 2014).

Increased LF has been observed in the brain of people suffering from HD (Tellez-Nagel et al., 1974; Goebel et al., 1978; Braak and Braak, 1992; Vonsattel and Difiglia, 1998). Thus, in HD brains, Braak and Braak found that the loss of neurons at the subiculum was marked by the presence of extraneuronal deposits of LF within layers of pyramidal neurons (Braak and Braak, 1992). Later, Vonsattel and Difiglia described that neurons from HD cases “contain more lipofuscin and may be smaller than usually expected” (Vonsattel and Difiglia, 1998).

Similarly, a transgenic mouse model for HD has shown that the formation of huntingtin neuronal intranuclear inclusions is accompanied by the appearance of accumulations of LF within the cytoplasm of neurons even in juvenile mice from 12 weeks old (Davies et al., 1997, 1999). Interestingly, Zheng and collaborators have demonstrated that the expression of full-length htt lacking its polyglutamine stretch (ΔQ-htt) in a mouse model of HD was able to rescue the HD phenotype, reducing huntingtin aggregates and LF levels, motor and behavioral deficits and extending life span (Zheng et al., 2010). The authors also found that ΔQ-htt expression increases autophagosome synthesis, arguing that this autophagy upregulation may be beneficial in diseases caused by toxic intracellular aggregate-prone proteins (Zheng et al., 2010), as it is the case of most neurodegenerative disorders. As we discussed previously, the observed normalization of LF levels may be directly correlated with autophagosome function restoration.

### Diabetic encephalopathy

Diabetic encephalopathy refers to a pathology that courses with brain damage associated with diabetes type 1 or type 2, caused by either acute hypoglycemia or severe hyperglycemia. In type 2 diabetes, diabetic encephalopathy increases the risk of developing AD and other forms of dementia, while in type 1 diabetic encephalopathy is less likely to develop into dementia as the patients are younger, but type 1 diabetics with encephalopathy are more likely to develop learning disabilities and deficits in memory retrieval. In a mouse model of type 1 diabetes induced by treatment with streptozotocin, Sugaya and collaborators found that diabetes onset caused accelerated accumulation of LF granules in trigeminal neurons (Sugaya et al., 2004). Later, Alvarez and collaborators demonstrated that in the same streptozotocin model, the animals showed a phenotype similar to diabetic encephalopathy with mild neurodegeneration in the dentate gyrus and a strong decrease in hippocampal proliferation. Interestingly, the authors found a marked enhancement of intracellular LF deposits, characteristic of increased oxidative stress and aging in both, the hilus and the subgranular zone and granular cell layer (Alvarez et al., 2009). In a different model of diabetes, induced by administration of alloxan, the animals also showed signs of diabetic encephalopathy with rapidly progressing neuronal and glial losses in the primary somatosensory cortex and hippocampus, accompanied by accumulation of LF in neocortical neurons (Volchegorskii et al., 2013). These findings with different mouse models of diabetes underscore the exquisite sensitivity of neuronal tissues to LF buildup, suggesting an active neurotoxic role of these aggregates.

### Other conditions

Aggregates of lipofuscin or ceroid LF are found in various metabolic disorders associated with mutations in several genes. Thus, in familial spastic paraplegia cases associated with neurodegeneration and gliosis, a marked presence of LF deposits in surviving neurons has been observed (Wakabayashi et al., 2001).

In rat cerebral cortex, cathepsin D (but no cathepsin B) has been found to colocalize with LF aggregates in the extracellular space (Jung et al., 1999) that in turn activates the p38 MAP kinase cascade through the increased intracellular generation of ROS, and promote the expression of iNOS and CAT-2, thereby inducing NO overproduction (Yamasaki et al., 2007). Cathepsin F-deficient mice (cat F-/-) display CNS neurons with large accumulations of lysosomal LF (Tang et al., 2006). Also in a murine model, it was observed that chronic sleep disruption resulted in frequent activation of wake-activated neurons (WAN), which promotes mitochondrial metabolic stress and increased LF accumulation in a subgroup of WAN (Zhu et al., 2015). On the other hand, chronic alcohol consumption induces increasing LF deposition in rat hippocampal neurons (Borges et al., 1986) and Purkinje (Lewandowska et al., 1994; Wenisch et al., 1997; Dlugos, 2015). Mice lacking the expression of glycerophosphodiester phosphodiesterase 2 (a six-transmembrane protein that cleaves glycosylphosphatidylinositol anchors) show early neurodegeneration with vacuolization, microgliosis, cytoskeletal accumulation, and LF deposition followed by astrogliosis and cell death (Cave et al., 2017).

As a whole, these data from human disorders and mouse models point to a common dyshomeostasis of the endolysosomal pathway not necessarily associated with oxidative stress and mitochondrial dysfunction, but in which LF is always present, likely acting as a pathology booster.

## Therapeutic approaches

As LF and other lipopigments, such as ceroids, represent a marker of post-mitotic normal and pathologic aging, and they have been causally associated with neuropathological changes, LF elimination has been suggested as a suitable target in anti-aging therapies (Riga et al., 2006b). Recently, a clinical trial (ClinicalTrials.gov identifier NCT00028262) for the treatment of patients with infantile NCL associated with mutations in the *CLN1* gene encoding for palmitoyl-protein thioesterase-1 has been carried out by orally administering the nucleophilic small molecules cysteamine bitartrate and N-acetylcysteine, aiming at the reduction of ceroid lipofuscin and granular osmiophilic deposits (GROD) (Levin et al., 2014). The results of the trial indicated that the patients presented a significant decrease of GROD in peripheral leukocytes, together with improved functional and behavioral parameters and fewer seizures. However, the results of these trials did not support the idea that the removal of the storage deposits correlates with a clinical benefit or halt neurodegeneration (Levin et al., 2014; Neverman et al., 2015).

There are also several approaches in the field of AMD targeting LF or its precursors. Thus, two visual cycle inhibitors, namely Fenretinide and Emixustat, have been used trying to prevent the phototoxicity and proinflammatory effects of LF. A phase II clinical trial with Fenretinide (NCT00429936) did not show efficacy to halt the growth rate of geographic atrophy (GA) in AMD, but patients seemed to tolerate it well. A phase IIa clinical trial with Emixustat (NCT01002950) showed a biological effect in GA in AMD patients. A Phase II/III study (NCT01802866) has been completed, but no results are yet available (Bandello et al., 2017) (information checked in May 2018).

A study with rats showed that treatment with melatonin or coenzyme Q10 for 4 weeks reduced the LF content of the hippocampus and carbonyl level (Abd El Mohsen et al., 2005). In fish, analysis of age-dependent mortality revealed that dietary restriction prevented the accumulation of LF in the liver and the neurodegeneration marker FluoroJade B in the brain (Terzibasi et al., 2009).

Regenerative neuroactive factors have been also suggested to enhance antioxidative defense, stimulate brain anabolism and potentiate lysosomal system, while decreasing LF levels. By acting also on glial cells, the mechanism of clearance of LF deposits and other residues appear to be also improved (Riga et al., 2006b).

Interestingly, the presence of LF appears to have a positive effect for certain therapies with antitumor drugs that produce free radicals, whose action is inhibitory or deadly for tumor cells (Schwarzburd and Aslanidi, 1991). Under the oxidative stress provoked by the drugs, it appears that normal cells respond with an increase in the amount of LF in their lysosomes and cytoplasm hampering further damage. Opposite, cancer cells, unable to accumulate LF particles are more susceptible to the action of the drug. In this sense, Schwartsburd in 1995 put forward the idea that lipofuscinogenesis is an adaptative cell response to oxidative stress that improves cell survival under harsh conditions (Schwartsburd, 1995). In *Drosophila melanogaster*, a similar protective effect of LF is suggested by *spin* gene mutants that presented degeneration of adult neural cells, while surviving neurons were atrophied with heavy LF deposition (Nakano et al., 2001). However, once a certain threshold of LF level is trespassed, and the system is overload, this mechanism appears to produce the opposite effect, by multiplying the effects of oxidative stress.

## Concluding remarks

As we grow older, LF gets accumulated in post-mitotic cells due to its highly complex cross-linked structure that is not amenable to degradation, as a marker of partially dysfunctional metabolism. Thus, LF has been traditionally considered a consequence of “normal” aging that gets increasingly deposited as cellular garbage, starting early in life.

**Figure 2:**
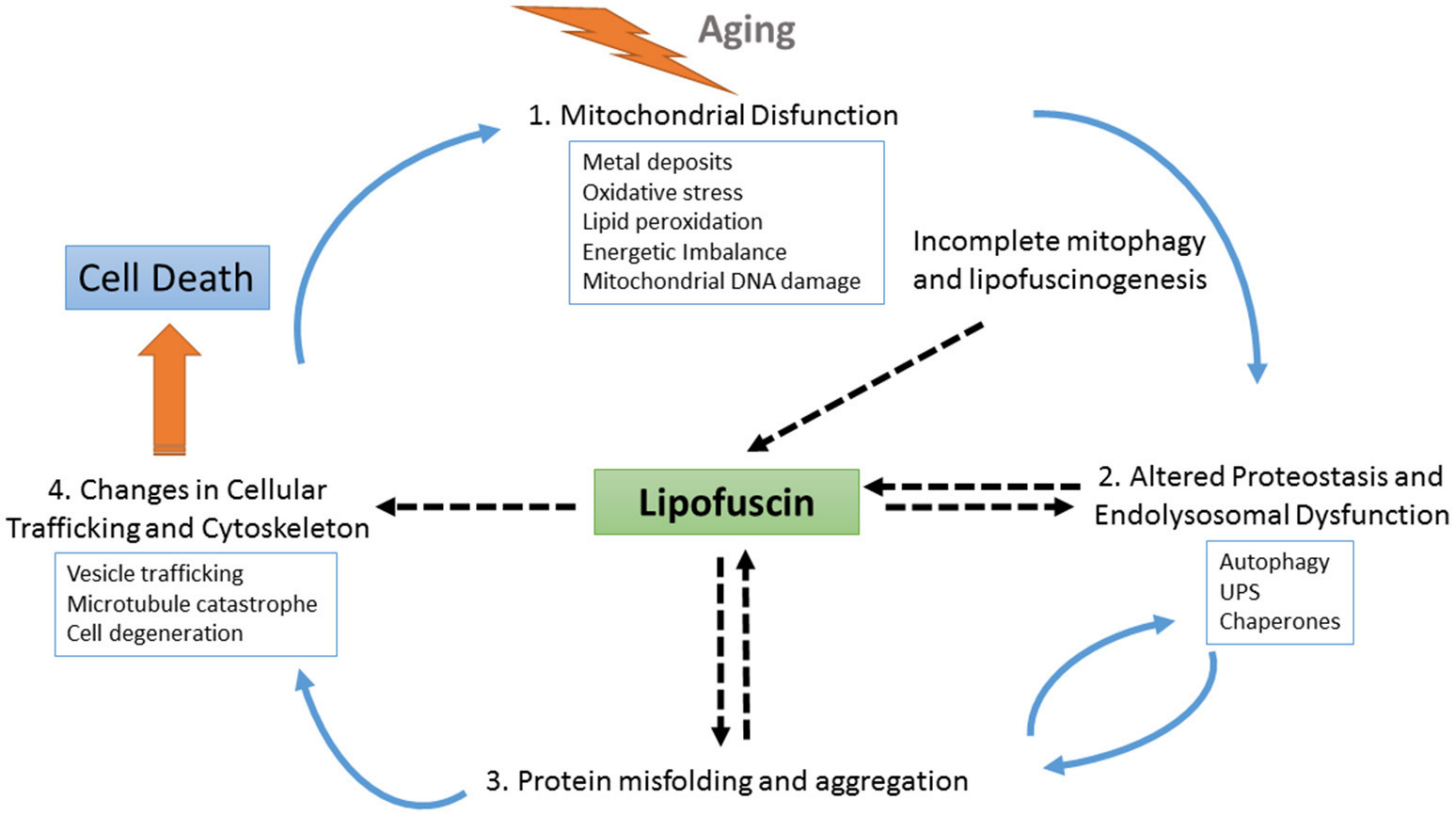
Schematic representation of the role of LF at different points of cellular physiology. With aging, the levels of oxidative stress increase, causing the impairment of several interconnected cellular homeostatic pathways such as the endolysosomal and mitochondrial systems and leading to defective proteostasis, incomplete mitophagy and the activation of lipofuscinogenesis. On the one hand, lipofuscin accumulation harms the proteostasis pathway, further promoting oxidative stress, energetic imbalance, and increased lipofuscinogenesis. On the other hand, altered proteostasis together with endolysosomal dysfunction also obstruct the clearance of misfolded proteins, which may accumulate as seeds for protein aggregation contributing to neurodegeneration. As protein aggregates deposits within the brain tissue, LF formation also increases, hindering both vesicle trafficking and cellular physiology. Finally, as the cell is unable to deal with the cascade of damaging events it degenerates. Upon cell death, LF content is released to the parenchyma, where it can be found associated with pathognomonic protein deposits, such as amyloid beta plaques. These LF deposits recruit cellular debris and metals ions, provoking more oxidative stress, and microglial activation, which triggers neuroinflammation and proinflammatory interleukin production, thus contributing to expanding the neurodegeneration.

Similarly, pathognomonic protein deposits such as amyloid beta plaques, tau neurofibrillary tangles or alpha-synuclein Lewy bodies are present even in normal individuals, and their accumulation appears to be the result of a protective cellular response to handle an excess of misfolded proteins (Ross and Poirier, 2005). Thus, the process of LF formation may represent a protective neuronal mechanism by which molecules that are highly toxic in soluble form are sequestered in a more stable, less-toxic state as aggregates (Zhao et al., 2011). However, recent findings indicate that LF is not an inert byproduct of cells, but it rather actively alters cellular metabolism at different levels by inhibiting the proteasome, impairing autophagy and lysosomal degradation, and serving as a reservoir of metal ions leading to ROS generation and apoptotic cell death (reviewed in Reeg and Grune, 2015) (see also Figure 2). Additionally, the dispersed nature of the deposits, which are distributed throughout the tissue, may argue for a mechanism of LF diffusion and seeding for new LF aggregates.

The presence of LF as a pathognomonic marker in both infantile-juvenile and adult neuronal ceroid lipofuscinoses, as well as its role as ROS amplifier mediated by UV-exposure in age-related macular degeneration, underscore the pathological role of LF in these groups of neurodegenerative disorders. However, the direct involvement of LF in other neurodegenerative disorders such as AD or PD is still a matter of debate.

In order to discuss whether LF is a subproduct of defective cellular homeostasis associated with aging or it has a pathological role of its own in neurodegeneration, it is relevant to compare the temporal profile of accumulation of LF aggregates with pathognomonic protein deposits associated with diverse neurodegenerative disorders. Interestingly, the temporal pattern of accumulation is similar to the one observed for protein deposits in different neurodegenerative disorders (Braak et al., 2011; Ferrer et al., 2011; Del Tredici and Braak, 2016). According to the study by Braak and coworkers, in the case of AD, both β-amyloid and neurofibrillar pathological features are present very early in life (even in the first decade), and also as in the case of LF, their presence increases with age (Braak et al., 2011). However, these pathology markers are not associated with overt disease until they reach a certain threshold that varies with age. Thus, lipofuscinogenesis and neuropathogenesis of the main CNS degenerative conditions seem to share an equivalent temporal kinetics: early pathological changes and slow relentless development with late sensorial, motor and cognitive deterioration.

Moreover, LF may also exert a neuropathological role by the dysregulation of protein homeostasis through its ability to sequester functional molecules and proteins (Hashemzadeh-Bonehi et al., 2006). Provided the hydrophobic nature of LF aggregates, a similar role sequestering other functional molecules and later release of diffusible molecules that are more neurotoxic cannot be ruled out. Additionally, the interaction with pathognomonic aggregation-prone proteins, such as Aβ, α-syn, or tau needs further research, but the presence of LF aggregates within neurodegeneration-specific protein deposits such as the AD senile plaques suggests an involvement of LF in pathogenesis (Cataldo et al., 1994; Giaccone et al., 2011) (see also Figure 1).

Taken together, all these data suggest a neuropathological role of LF by impairing the same mechanisms and acting like other protein aggregates (e.g., amyloid beta, tau, alpha-synuclein) of different neurodegenerative diseases. Thus, we argue that LF plays a transversal function on both cellular senescence and across neurodegeneration. Provided that LF increasingly accumulates with age, and assuming the hypothesis that LF is a risk factor for different neurodegenerative disorders, one may expect higher incidences of neurodegenerative disorders with the increased levels of the risk factor (in this case LF). However, although age is also one of the strongest risk factors for late-onset neurodegenerative disorders, the normalized incidence of each entity (perhaps with the exception of AD) peaks at certain age range, from which it starts to decay. This apparent disagreement between a continuously increasing buildup of LF deposits as a risk factor and the variable age-specific peak incidence of diverse neurodegenerative disorders (de Pedro-Cuesta et al., 2015) is similar to other pathognomonic protein deposits. This could be explained either by a subclinical expression of LF deposits (vs. the clinical definition of incidence counts) or by more complex phenomena associated with a ceiling effect, above which the LF levels are already irrelevant.

In line with the work from Giaccone and collaborators that discussed the potential pathogenic role of LF in AD (Giaccone et al., 2011), we propose that LF is one relevant effector of aging that represents a risk factor (or more properly, a risk modifier) for neurodegenerative disorders. In the context of our previous works, LF fits well with the concept of *driver* (defined as “neither protein/gene nor entity-specific features identifiable in the clinical and general epidemiology of neurodegenerative diseases as potential footprints of templating/spread/transfer mechanisms”) (de Pedro-Cuesta et al., 2016a). This transversal approach provides a unified view of LF pathological role in neurodegeneration for genetic (neuronal ceroid lipofuscinosis, and others) and sporadic neurodegenerative disorders with specific age-at-onset related patterns (de Pedro-Cuesta et al., 2016b). In conclusion, the relentless accumulation of LF is clearly associated to aging, but as we have reviewed herein, increasing evidence suggest that LF formation leads to neuropathology exacerbation by different mechanisms (see Figure 2). Further studies to determine whether LF accumulation itself, both within the neuronal cell and in the brain parenchyma, is contributing to neuronal loss and disease are needed. Proof-of-concept studies in mouse models aiming at specifically clearing existing LF deposits in NCLs may help to elucidate the role of LF and guide new therapeutic approaches for neurodegenerative disorders.

## Author contributions

All authors participated in the conception and aims of the review. AM-G, AK, and OC participated in the literature search, and analysis of references, participated in the experimental acquisition of images for Figure 1, wrote sections of the manuscript. MM performed the critical revision of the article. MC wrote the first draft of the manuscript. All authors contributed to manuscript revision, read and approved the submitted version.

### Conflict of interest statement

The authors declare that the research was conducted in the absence of any commercial or financial relationships that could be construed as a potential conflict of interest.
